# Hedging Your Bets: Intermediate Movements as Optimal Behavior in the Context of an Incomplete Decision

**DOI:** 10.1371/journal.pcbi.1004171

**Published:** 2015-03-30

**Authors:** Adrian M. Haith, David M. Huberdeau, John W. Krakauer

**Affiliations:** 1 Department of Neurology, Johns Hopkins University, Baltimore, Maryland, United States of America; 2 Department of Biomedical Engineering, Johns Hopkins University, Baltimore, Maryland, United States of America; 3 Department of Neuroscience, Johns Hopkins University, Baltimore, Maryland, United States of America; Harvard University, UNITED STATES

## Abstract

Existing theories of movement planning suggest that it takes time to select and prepare the actions required to achieve a given goal. These theories often appeal to circumstances where planning apparently goes awry. For instance, if reaction times are forced to be very low, movement trajectories are often directed between two potential targets. These intermediate movements are generally interpreted as errors of movement planning, arising either from planning being incomplete or from parallel movement plans interfering with one another. Here we present an alternative view: that intermediate movements reflect uncertainty about movement goals. We show how intermediate movements are predicted by an optimal feedback control model that incorporates an ongoing decision about movement goals. According to this view, intermediate movements reflect an exploitation of compatibility between goals. Consequently, reducing the compatibility between goals should reduce the incidence of intermediate movements. In human subjects, we varied the compatibility between potential movement goals in two distinct ways: by varying the spatial separation between targets and by introducing a virtual barrier constraining trajectories to the target and penalizing intermediate movements. In both cases we found that decreasing goal compatibility led to a decreasing incidence of intermediate movements. Our results and theory suggest a more integrated view of decision-making and movement planning in which the primary bottleneck to generating a movement is deciding upon task goals. Determining how to move to achieve a given goal is rapid and automatic.

## Author Summary

Two critical processes need to occur before a movement can be made: identification of the goal of the movement and selection and preparation of the motor commands that will be sent to muscles to generate the movement—in other words, what movement to make, and how to make it. It has long been thought that preparing motor commands is a time-consuming process, and theories advocating this view have pointed to instances where apparently the wrong motor commands are issued if insufficient time is available to prepare them. The usual pattern of these wayward movements is that they are intermediate between two potential targets. In this article we show how such intermediate movements can alternatively be viewed as reflecting an intelligent and deliberate decision about how to move, given uncertainty about task goals. Our theory is supported by experiments that show that intermediate movements only occur in conditions where they are advantageous. The implication of our theory is that the primary bottleneck to generating a movement is deciding on exactly what to do; deciding how to do it is rapid and automatic.

## Introduction

In the reaction time before a movement is initiated, two distinct processes are thought to occur: first, the exact goals of the movement must be decided upon and, second, the actions that will achieve the chosen goal must be selected and/or prepared [1]. Decisions about high-level movement goals have been well-characterized in terms of an accumulation of sensory evidence over time [2,3]. The process of selecting and/or preparing the actions to achieve a chosen goal, which we refer to here as *movement planning*, is classically thought to require further time-consuming computations [4,5,6]. The relative contribution of goal selection and movement planning to the reaction time remains a matter of considerable debate [7,8].

One way to study the process of movement planning is to interrupt it and examine the behavioral consequences. When a reaching movement is released at a lower-than-normal reaction time, movements appear to be biased away from the target stimulus towards a ‘default’ movement [4,9,10]. As preparation time increases, movements gradually converge on the target. Similar intermediate movements are observed if a target jumps shortly before movement onset [11,12,13,14,15] or in tasks that either deliberately or inadvertently create ambiguity about task goals [16,17,18,19]. These intermediate movements have been variously interpreted as reflecting incomplete movement planning or interference between parallel plans to each potential goal. Either interpretation suggests that intermediate movements occur as an unintentional artifact of stressing an underlying planning mechanism.

Although generally well accepted, these existing interpretations of intermediate movements are at odds with more contemporary theories of movement execution based on optimal control theory [20]. According to this theory, a single, flexible feedback control policy can be sufficient to generate a wide variety of movements and rapidly switch between them based on new sensory observations [21,22]. Such an organization dispenses with the need to re-plan a movement each time the movement goal changes and is incompatible with replanning-based explanations for intermediate movements. Here we show how intermediate movements can be understood within an optimal control framework if the control policy takes into account an evolving decision about the location of movement goals. Our theory, therefore, frames intermediate movements as reflecting a deliberate plan to deal with uncertainty about movement goals, rather than as resulting from erroneous movement planning.

A critical prediction of this theory is that intermediate movements should only occur when potential movement goals are compatible, i.e. when they require kinematically similar movements. Given compatible goals, an intermediate movement can bring the hand closer to both goals simultaneously, pending the arrival of further information about which goal to ultimately commit to [23,24]. If the compatibility between goals is eliminated, either by separating the goals more widely in space [4,25] or by imposing an obstacle between them, intermediate movements no longer offer this advantage and all movements should instead be directed to one target or another. We verified these predictions in three experiments that varied the compatibility between movement goals in two distinct ways.

## Results

We propose an alternative interpretation of intermediate movements: that they reflect an optimal and deliberate movement plan given uncertainty about task goals. When potential movement goals are compatible with one another, intermediate movements allow commitment to a specific goal to be deferred until further evidence can be acquired after movement has begun. A specific prediction of this theory is that reducing the compatibility between potential goals will reduce the incidence of intermediate movements. We tested this hypothesis in a target-jump paradigm by varying the compatibility between pre-jump and post-jump goals. In Experiments 1 and 2, we reduced goal compatibility by increasing the angular separation between target locations from 45° to 90° to 135°. In Experiment 3, we reduced goal compatibility by introducing a virtual barrier between targets spaced 45° apart.

### Experiment 1: The incidence of intermediate movements depends on target jump amplitude

**Fig 1:**
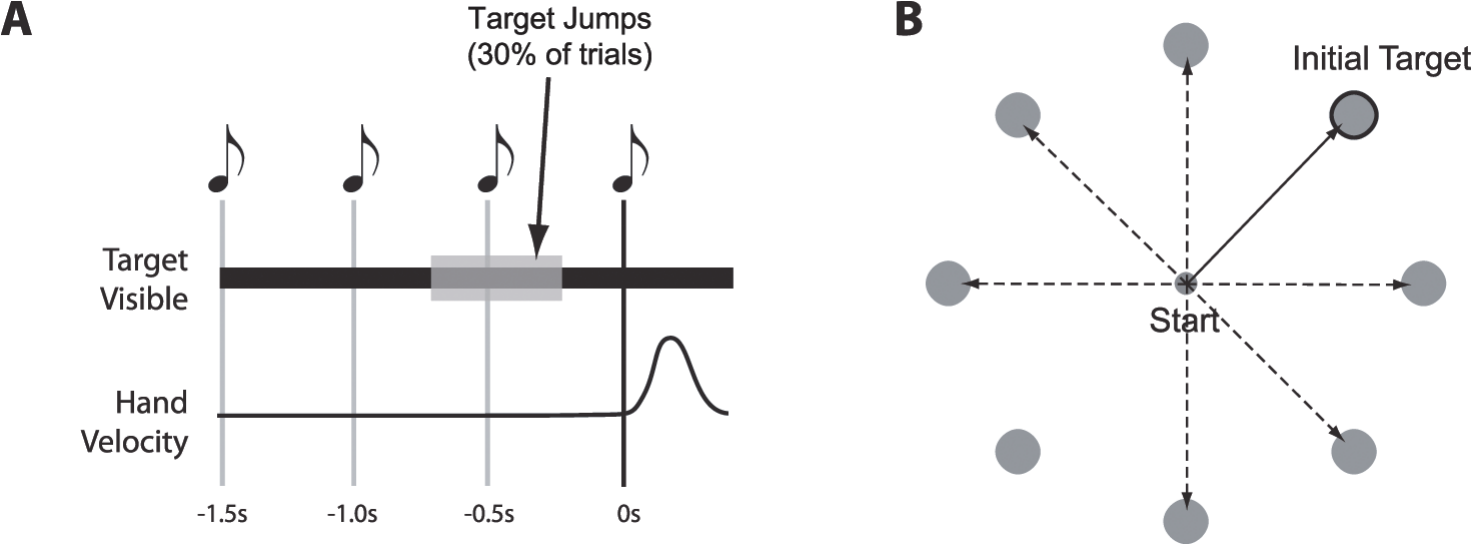
Experiment 1 setup. A) Subjects were trained to initiate movement synchronous with the last of four tones played at 500ms intervals. B) Subjects made center-out movements to “shoot” through one of 8 equally spaced targets. The target appeared synchronously with the first tone. On 30% of trials, the target was jumped by ±45°, ±90° or ±135° at a random time between 150 and 550ms before the fourth (i.e. final) tone. Solid arrow/bold target indicates movement required before target jump. Dashed arrows indicate potential movements required after a target jump.

Subjects were trained to initiate their movements at a precise time during each trial (Fig. 1A) and make center-out movements to “shoot” through a target. On a subset (30%) of trials, the target jumped by ±45°, ±90° or ±135° at an unpredictable time (between 150ms and 550ms) prior to movement initiation (Fig. 1B). We were interested in how subjects’ behavior (specifically, the initial direction of their movement) depended on the amount of re-preparation time (rPT) available. That is, the amount of time that elapsed between when the target jumped and when the subject initiated their movement.

**Fig 2:**
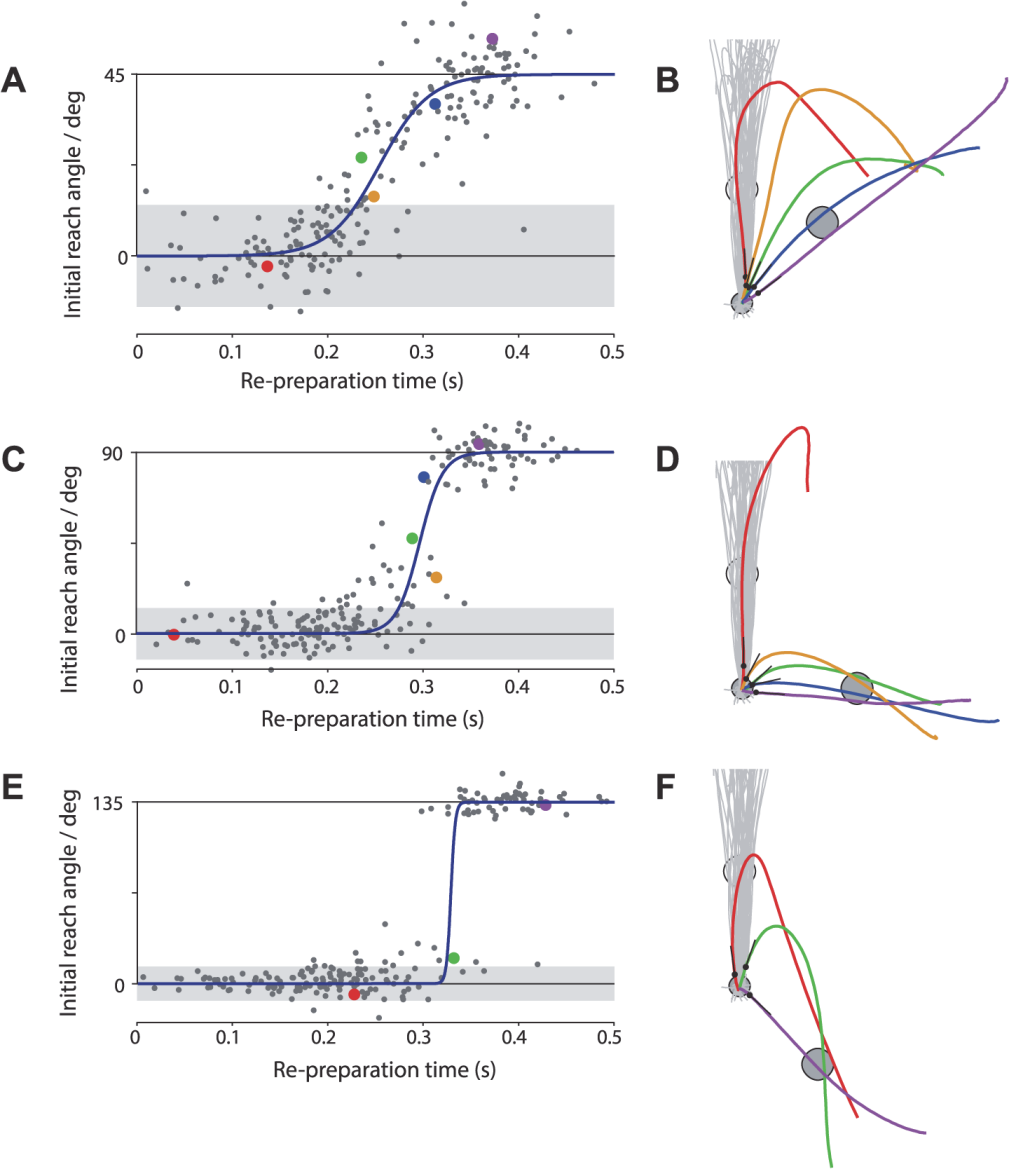
Behavior of a representative subject in Experiment 1. Left axes show the relationship between available re-preparation time (equal to the delay between target jump time and movement onset) and initial reach direction for 45° (A), 90° (C) and 135° (E) jumps. Each point represents a single jump trial. Solid lines indicate sigmoidal fits obtained by maximum likelihood estimation (see Methods). Shaded region indicates 95% confidence interval (1.96×standard deviation) for movement directions observed on non-jump trials. Right panels: trajectories of selected movements that were initiated in a direction intermediate between the original and post-jump target location following 45° (B), 90° (D) and 135° (F) jumps. Line colors indicate data point in corresponding plots to the left. Black circles indicate the position 100ms after movement onset at which point trajectory direction was calculated based on tangential velocity (adjoining black line). The original (pre-jump) target is located at the 12 o’clock position. Gray trajectories illustrate a sample of trajectories from non-jump trials.

**Fig 3:**
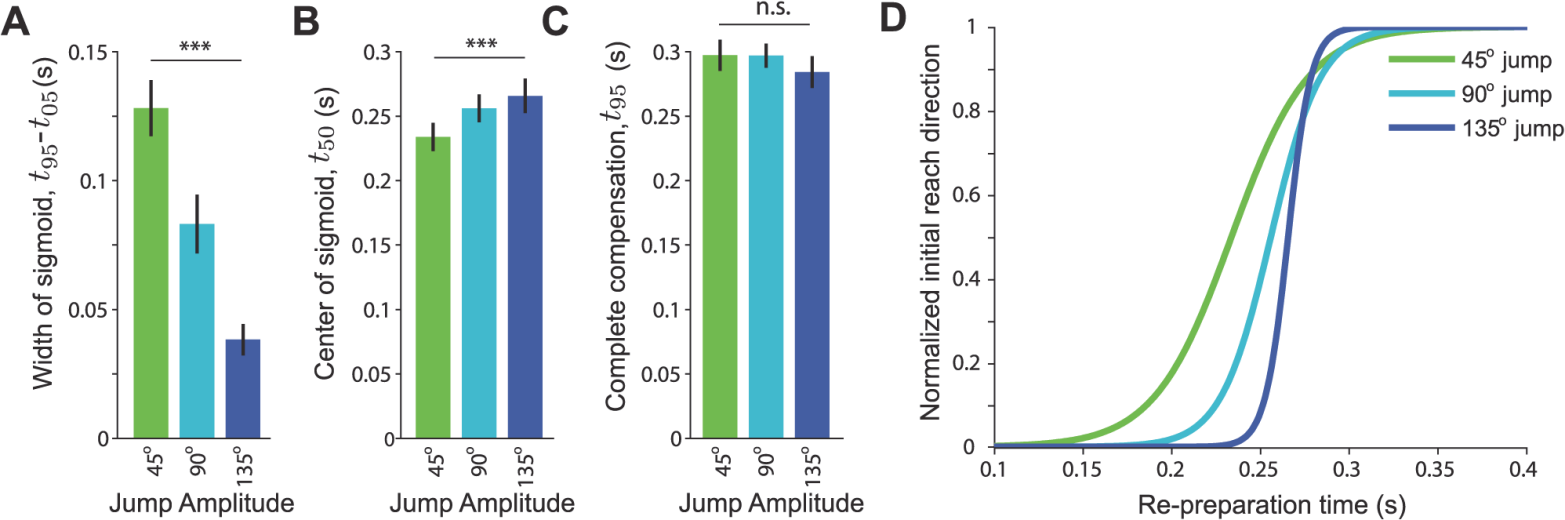
Group results for Experiment 1. Estimated sigmoid parameters across all subjects for each target jump amplitude. A) Total time over which reach direction varied (*t*_95_–*t*_05_; proportional to slope parameter, *τ*). B) Center of sigmoid, *t*_50_. C) Time required to fully compensate for the target jump, *t*_95_. Error bars indicate s.e.m. D) Average sigmoidal fits to behavior across all subjects obtained by averaging parameters *τ* and *t*_50_.

Fig. 2 shows data from a representative subject. On trials in which the target jumped by 45°, we observed a continuous relationship between rPT and initial reach angle (Fig. 2A,B). For movements initiated less than 200ms after the target jump, movements were directed towards the original target location. Between 200ms and 350ms, the initial reach angle changed gradually from the original to the new target direction as the rPT increased. For movements initiated more than 350ms after the target jump, this subject was consistently able to compensate for the change in target location. A similar pattern held for the behavior in response to 90° jumps, only that the subject adhered to the initial reach direction slightly longer and the transition between targets was steeper (Fig. 2C,D). For the largest target jumps (135° change in reach direction), there was no clear transitional period between targets. Instead, behavior switched abruptly at around 350ms from movements directed towards the initial target to movements directed towards the post-jump target (Fig 2E,F). All subjects showed the same qualitative pattern of gradual adjustment of reach direction for small target jumps, and more abrupt adjustment for larger target jumps. Interestingly, there were still a small number of intermediate movements generated following large target jumps (Fig. 2 E,F), suggesting a continuous underlying change in reaching direction, albeit so rapid that the transition appeared abrupt. To characterize this behavior quantitatively, we fit sigmoid functions to the relationship between rPT and initial reach direction (see Methods, Eq. (2)). This yielded two parameters for each subject, for each jump amplitude. The first parameter, *τ*, reflects the timescale over which this transition occurred. The second parameter, *t*_50_ (s) reflects the latency of the change in initial movement direction. Comparing the fitted sigmoid parameters for all subjects, we found that the slope of the sigmoid, *τ* differed significantly across jump amplitudes (F(2,18) = 22.6; p<0.0001) (Fig. 3A). *Post-hoc* pairwise comparisons of *τ* across jump amplitudes were all significant (p<0.05). Thus the transition in initial reach direction was consistently more abrupt for large amplitude target jumps than for smaller amplitude jumps, confirming our predictions.

Next, we examined the latency of compensation for the target jump. We found that *t*_50_, the time at which subjects would make an exactly intermediate movement, also depended on jump amplitude (F(2,18) = 11.96; p<0.001) (Fig. 3B). Visual inspection of the data suggests that the re-preparation time required for complete compensation, i.e. the shortest delay at which movements directed to the post-jump target were reliably observed, seemed to be consistent across jump amplitudes. To test this hypothesis, we considered the time *t*_95_ at which each fitted sigmoid reached 95% compensation. This measure showed no significant difference across jump amplitudes (F(2,18) = 1.3; p = 0.30) (Fig. 3C). Conversely, differences in the times at which compensation began (*t*_05_; 5% of sigmoid height) were highly significant across subjects (F(2,18) = 32.04; p<.0001). Thus, target jumps of larger amplitude angles did not require a longer period of re-preparation, despite requiring a larger change in movement direction. The primary difference in behavior across jump amplitudes was in the time at which the change in target location began to be reflected in subjects’ movements.

Despite extensive training, all subjects exhibited considerable variability in their movement initiation times. The standard deviation in movement initiation time, averaged across subjects, was 79±21ms. This value was quite large when compared with the timescales over which subjects’ behavior changed (∼100ms). We considered whether the delay between the target jump and the *intended* time of movement initiation (i.e. the delay between the target jump and the fourth tone) could serve as a better predictor of behavior than rPT. To test this possibility, we repeated our analysis using the absolute time of the target jump instead of the delay between target jump and movement onset (rPT). The total log-likelihood of the sigmoid fit (including data points classified as outliers) was significantly worse (F(1,6) = 48.2; p<0.001)). Thus we can conclude that behavior depended specifically on the actual time of movement initiation and not on the intended time of movement initiation.

### Experiment 2: The time course of compensation for a target jump does not depend on target uncertainty

**Fig 4:**
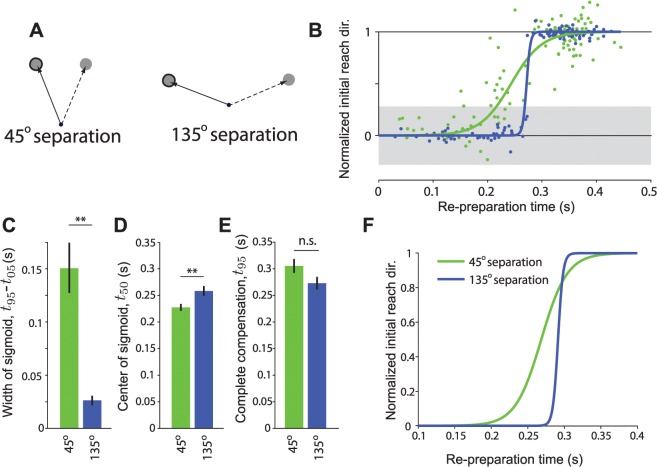
Experiment 2 results. A) Only two target locations were possible within each block, either separated by 45°, or 135°. The target was jumped on 30% of trials. Solid arrow/bold target indicates movement required before target jump. Dashed arrows indicate potential movements required after a target jump. B) Behavior in jump trials and sigmoidal fits trials for a representative subject (green = 45° jump; blue = 135° jump). C)—E) Estimated sigmoid parameters across subjects (as Fig. 3A-C). F) Average sigmoid fits to behavior across all subjects.

An important feature of Experiment 1 is that although the disappearance of the initial target is unambiguous, the target could have jumped to a number of different locations. We performed a second experiment to determine to what degree, if any, this ambiguity affected the timecourse of compensation for the jump. In Experiment 2, the target only appeared in two possible locations within each block (Fig. 4), such that whenever the target jumped, the location it jumped to was always known unambiguously.

Despite the difference in paradigms, subject behavior was identical to that seen in Experiment 1. The initial reach direction changed gradually as a function of rPT for a 45° separation between targets, but changed abruptly when the targets were separated by 135°. As in Experiment 1, the extent of intermediate movements (slope of the sigmoid) differed significantly across jump amplitudes (F(1,5) = 24.66, p<0.01), as did the latency, *t*_50_ (F(1,5) = 36.2, p<.01). The time at which compensation was complete, *t*_95_, was similar across target separations, although appeared to be slightly earlier for the 135° than 45° separation (F(1,5) = 5.62, p = 0.06). We can conclude from the results of Experiment 2 that ambiguity about the precise location of the post-jump target did not significantly influence behavior.

### Experiment 3: Intermediate movements are suppressed when goals are incompatible

In Experiments 1 and 2, we showed that decreasing compatibility between goals by increasing their angular separation led to a more rapid transition between movement directions, with a corresponding decrease in the incidence of intermediate movements. This result is consistent with our hypothesis that intermediate movements constitute an intelligent solution to the problem of moving amid goal uncertainty. However, varying the jump amplitude also affected the degree of similarity between the motor commands required before and after the jump. Intermediate movements may have emerged from interference between overlapping movement representations, rather than because of an exploitation of task-level compatibility between goals. In Experiment 3, we controlled for this possibility by using an alternative approach to reducing the compatibility between the pre-jump and post-jump targets that allowed us to vary the compatibility between goals while keeping the pre-jump and post-jump goals consistent across conditions.

**Fig 5:**
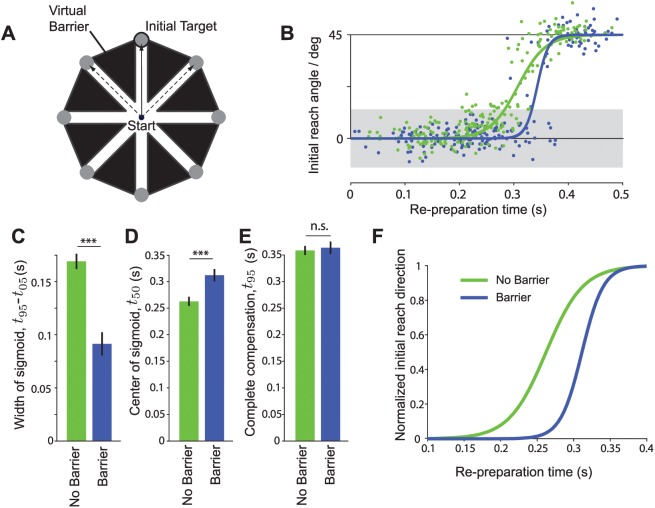
Experiment 3 results. A) The experimental setup was as experiment 1 (Fig. 1A), except that in one condition a series of virtual barriers was put in place to penalize subjects for making intermediate movements. In this experiment, the target only jumped by ±45°, and did so on 30% of trials. Solid arrow/bold target indicates movement required before target jump. Dashed arrows indicate potential movements required after a target jump. B) Behavior on jump trials for a representative subject. Blue points indicate individual trials from the session in which barriers were present. Green points indicate individual trials in which the barriers were absent. Solid lines indicate sigmoidal fit. Shaded region indicates 95% confidence interval for movement direction based on trials from the no-barriers session in which the target did not jump. C)—E) Estimated sigmoid parameters across subjects (as Fig. 3A-C). F) Average sigmoid fit to behavior across all subjects following a target jump.

We created a series of virtual barriers between adjacent targets (Fig. 5A). Any intermediate movements were penalized by playing an unpleasant rasping tone and withholding points and other success cues from subjects on trials in which they collided with the barrier. We tested the behavior of subjects in response to target jumps of 45° amplitude both with and without these barriers present (Fig. 5B). As before, when the barriers were not present, subjects exhibited a gradual change in movement direction with a large number of intermediate movements. With the barriers present, however, instead of this gradual change in behavior, subjects switched more abruptly from one direction to another. Applying the same analysis as in Experiment 1, we found a significant difference in the timescales of the change in initial movement direction, *τ*, across conditions (F(1,7) = 30.75; *p*<.001) and also in the latency *t*_50_ (F(1,7) = 29.98; *p*<.0001). The time required to fully compensate for the target jump (*t*_95_) was not significantly different across conditions (F(1,7) = 2.57; *p* = 0.15). Importantly, differences in behavior between the barrier and no barrier conditions cannot be attributed to an increase in accuracy demands when barriers were present: variability in initial reach direction on non-jump trials was not strongly affected by the presence of a barrier (s.d. in initial reach direction without barrier = 4.8±.6°, with barrier = 4.4±.9°; F(1,7) = 3.58; *p* = 0.1).

Overall, we found that the behavior following 45° jumps with barriers was qualitatively similar to that observed for 135° jumps in Experiment 1 when movement paths were unconstrained. This confirmed our hypothesis that goal compatibility, and not the magnitude of difference in required movement directions, was the key determinant of behavior following a target jump.

### Abrupt and gradual shifts in reach direction as a consequence of optimal control under goal uncertainty

Our experimental results showed a clear pattern whereby the timecourse of adjustment in behavior following a target jump, and, consequently, the incidence of intermediate movements, dependeds upon the compatibility between the pre-jump and post-jump goals. For nearby targets, which are highly compatible, initial movement direction was adjusted gradually as a function of available re-preparation time. When the potential movement goals were incompatible with one another, initial movement direction switched abruptly at a clear threshold rPT.

**Fig 6:**
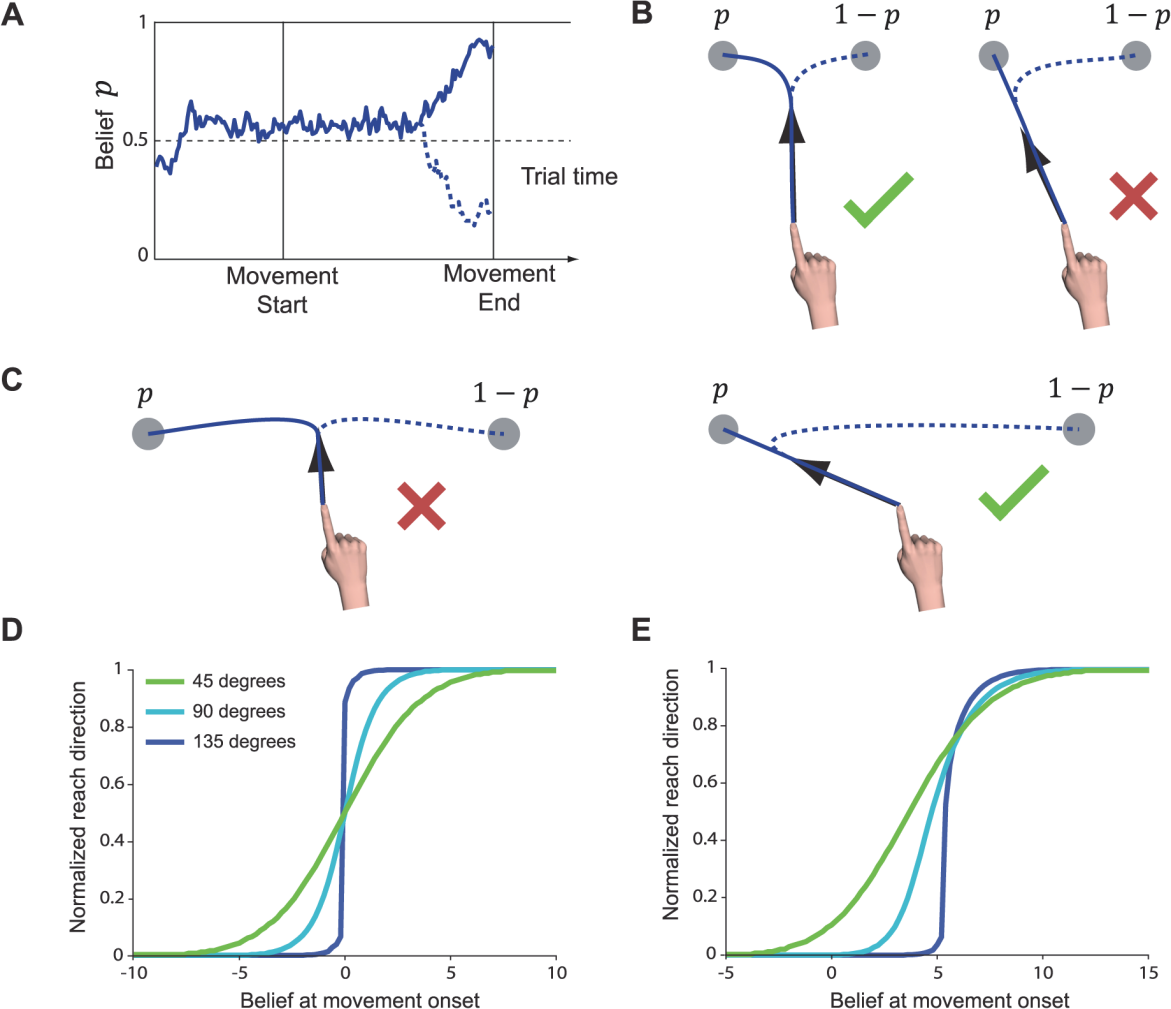
Computational model. A) Subjects are uncertain as to the true goal location among two possibilities and expect their beliefs to vary over time. In this case, the subject marginally favors the leftward target at movement onset (*p*_0_ > ½). During movement, this belief may either strengthen (solid line) or reverse (dashed line). B) When targets are nearby, the optimal course of action is to bias movement only slightly towards the more likely target, allowing greater flexibility later in the movement. C) When targets are widely separated, an intermediate movement is less advantageous. Instead, the optimal course of action is to commit to the more likely target from the outset. D) Predicted initial reach direction (normalized so that 0 corresponds to initial target, and 1 corresponds to the post-jump target) is plotted for a variety of target separations as a function of the initial belief state *r*_0_. E) As D), but for a model that uses an asymmetric cost function (see Methods).

We formalized our intuition about these results through a mathematical model (Fig. 6). We suppose that, in the immediate aftermath of the target jump, the subject must determine based on sensory information whether the target has jumped and, if so, where it has jumped to. Although information about the target was presented discretely and unambiguously, the detection of such unambiguous stimuli still may entail significant uncertainty. We modeled this simple decision about the location of the target as a process of noisy evidence accumulation. Critically, this implies that subjects were transiently uncertain as to the true location of the target, but became more confident as more time elapsed following the target jump.

We combined this decision-making process with an optimal control model of movement generation. We assume that each potential target is associated with some accuracy cost *J*_*x*_ that rewards movements that pass through the goal region and penalizes movements that do not (see Methods). We augment the usual state of the motor apparatus, **x**_*t*_, with a dynamic stochastic variable *r*_*t*_ reflecting accumulating evidence about the true identity of the target. Paralleling standard models of decision-making [3,26], *r*_*t*_ represents the log odds ratio of the belief *p*_*t*_ that the initial target is the true target location: $r_{t}=\log\left(\frac{p_{t}}{1-p_{t}}\right)$. *r*_*t*_ is allowed to vary from −∞ (certain that the target has not jumped) to +∞ (certain that the target has jumped). Together with an effort cost *J*_**u**_, the overall expected endpoint cost is then given by a sum of the accuracy costs associated with each target, weighted by their beliefs (Equation 9). Optimizing this cost yields a single, fixed control policy **u**_*t*_ = *π*(**x**_*t*_,*r*_*t*_,*t*) that guides responses to fluctuations in both the state of the plant and beliefs about the target. In principle this model could be used to predict full movement trajectories. However, the critical prediction of interest is the decision about what direction to move in at the very start of the movement given the prospect of gaining further evidence later on during movement i.e. whether or not an intermediate movement should be generated.

A general solution for this class of control problem is intractable for high-dimensional plants due to the non-quadratic form of the cost function. We therefore examined the behavior predicted by this theory in a simplified one-dimensional model of the center-out reaching task. In this model, the optimal initial reach direction is driven solely by the belief state at movement onset, *r*_0_, and the compatibility between potential goals. We cannot precisely know the timecourse of changes in belief in subjects following the target jump (i.e. how *r*_0_ varies as a function of time since the target jump). However, we assume that the change in belief should follow the same monotonic timecourse regardless of the size of the target jump. Thus the model explains differences in behavior across different jump amplitudes as being due to there being different optimal initial reach directions associated with similar belief states. Our aim in the model was to demonstrate that decreasing the compatibility between goals (by increasing the distance between them) leads to more abrupt changes in behavior as a function of preparation time. Fig. 6D illustrates the predicted initial reach direction in this simplified model as a function of the belief about the target location at movement onset. As can be clearly seen, the sensitivity of behavior to the belief at the time of movement onset depends significantly on the separation between targets. Our theory also naturally explains the absence of intermediate movements in the presence of a barrier, as seen in Experiment 3. The presence of the barrier reduces the decision about initial reach direction to a discrete choice, in which case the subject should select the most likely target direction at the time of movement onset, leading to step-like behavior.

Although the simulations clearly demonstrate the relationship between goal compatibility and the timescale of the change in reach direction, our simulations also reveal an interesting discrepancy between the theory and the data. In the model, behavior across all target jump amplitudes is aligned for exactly halfway intermediate movements, i.e. where *r*_0_ = 0. Consequently, large-amplitude target jumps are predicted to be fully compensated earlier than small-amplitude jumps. In the data, however, we observed that full compensation for the target jump occurred at similar delays across all jump amplitudes. One potential explanation for this is that in the model, the pre-jump and post-jump targets are treated symmetrically. The fact that the target jumped on only a subset of trials may have biased subjects towards the initial target. Subjects may even possess an innate bias towards their original movement plan in the event that circumstances unexpectedly change. Asymmetry may also have arisen from differences in confidence in the exact target location between pre- and post-jump targets (although the results of Experiment 2 allow us to largely rule out this possibility). There are therefore a variety of asymmetries between the pre and post-jump targets that are not captured by the basic model. We attempted to accommodate these effects within the model through an asymmetric cost function which effectively penalized a miss more heavily when the true target turns out to be the original target. We found that imposing such an asymmetric cost was able to better reproduce the observed pattern of behavior (Fig. 6E). While admittedly post-hoc, these results demonstrate that the basic modeling framework can feasibly be extended to account for this aspect of the data.

## Discussion

Existing theories have proposed that intermediate movements emerge as an artifact of low-level planning mechanisms stressed by the presence of multiple targets [17,27]. Here, we have presented an alternative, normative explanation for the existence of intermediate movements. Our theory, similar to previous arguments by Hudson, Landy and Maloney [24], suggests that intermediate movements are in fact goal-directed and reflect an attempt to optimize performance amid ambiguity about task goals. Our experiments verified an important implication of this theory: that the existence of intermediate movements should depend on compatibility between potential movement goals. In particular, in Experiment 3, we found that a change in high-level task requirements (avoiding a visual obstacle) was sufficient to eliminate the occurrence of intermediate movements that are normally seen following a 45° target jump.

Accounting for this result presents a challenge for low-level mechanistic theories of intermediate movements. Unlike a normative model, they do not naturally predict that the presence of intermediate movements should be dictated by task-level constraints. Instead they would need to invoke additional mechanisms that can suppress intermediate movements when this is necessary. Positing such mechanisms, however, raises the question of why intermediate movements should ever occur if they can be so easily suppressed. Only a normative explanation can satisfactorily resolve this question.

An important implication of our general theory is that movement planning—selecting and preparing the actions required to achieve a specified goal—may occur without any need for additional computation. We suggest that the primary bottleneck to generating a movement is determining the appropriate course of action [1] and that movement itself is generated according to a fixed, cached control policy. This idea is supported by our observation of abrupt switching between movement directions. Similar abrupt switching of behavior based on new sensory information has been reported in the context of saccades [28,29] and obstacle avoidance with the hand [22]. Abrupt switches in the direction of a saccadic eye movement have previously been interpreted as reflecting a very rapidly made decision [8]. Our theory suggests an alternative interpretation: that the timecourse of the underlying decision process may in fact be much slower, but the time of the switch reflects a tipping point at which the accumulated evidence favors the new target over the old one. Eye movements also exhibit intermediate movements in the presence of distractors [15,30,31], usually referred to as ‘averaging saccades’. We suggest that our theory also offers a potential normative explanation for this phenomenon, with the caveat that new information coming to light must be registered through a corrective saccade after termination of the primary saccade, as opposed to online corrections mid-movement.

### Potential alternative explanations for intermediate movements

Our experiments extend and reinterpret a classic series of studies by Ghez and colleagues, who developed the timed-response paradigm to examine movement planning and preparation [4,9,32,33]. In their experiments, only two movement directions were possible in each trial (as opposed to 8 in our experiments) and ambiguity was created by providing no target information at all until shortly before movement onset (as opposed to jumping an existing target as in our experiments). Their results were qualitatively similar to our own, with intermediate movements occurring at low preparation times when targets were narrowly separated but not when widely separated. An advantage of our target-jump approach is that we were able to control the initial belief state of the subject which, along with the low proportion of jump trials and multiple potential targets, made it unlikely that intermediate movements were the result of subjects adopting a deliberate aiming or guessing strategy. Our experimental results therefore reinforce the view that intermediate movements seen at low preparation times reflect an implicit property of the motor system rather than an explicit strategy.

Ghez and colleagues suggested that intermediate movements at narrow separations were due to incomplete specification (i.e. preparation) of the motor commands required for movement. This theory cannot, however, explain why intermediate movements do not occur for more widely separated targets or in the presence of a barrier. Ghez and colleagues therefore suggested the existence of two distinct mechanisms of movement planning: a discrete mechanism responsible for the abrupt behavior seen at wider separations and a continuous re-specification mechanism operating at more narrow separations (see also [25]). Our theory offers a more satisfying and parsimonious explanation for these contrasting modes of behavior: that they reflect qualitatively different solution regimes to the same optimization problem.

Some authors have suggested that intermediate movements following a target jump occur because the target is perceived to be at a location intermediate between goal locations [12,13]. Similarly, intermediate movements could potentially reflect an interpolation between movement plans, rather than between perceived goal locations. In either case, such interpolation mechanisms can plausibly account for the pattern of intermediate movements following small (45°) target jumps, and would also predict an abrupt switch in reach directions following 180° jumps. However, interpolation would predict only a relatively modest change in the pattern of intermediate movements as the target separation increased from 45° to 90° to 135°. In particular, this kind of model predicts a far smaller difference in behavior between the 45° case and the 135° case than suggested by our data. In particular, individual subjects tended to show an abrupt transition between movement direction following 135° jumps (e.g. Fig. 2E). This abrupt transition is consistent with the findings of Ghez et al. [4] who reported no intermediate movements when potential targets were separated by 120°. A model based on a direct interpolation between movement plans or goals does not predict such an abrupt transition until the jump amplitude becomes close to 180° and cannot therefore fully account for our findings.

Intermediate movements are often interpreted as evidence for interference between parallel movement plans. Tasks that directly manipulate goal uncertainty, either by delaying disclosure of goal information [4,16,17], by presenting distractors [18], or by providing deliberately ambiguous cues [34], yield intermediate movements. More abstract cognitive decisions can have a similar effect [19]. In all cases, ambiguity about the goals of the movement is believed to lead to interference between associated movement plans, which ultimately leads to errant intermediate movements being generated. The existence of such intermediate movements is therefore thought to offer insights into the underlying mechanism of movement planning. One would expect that such interference, arising from low-level mechanisms, should be unavoidable. This is, however, inconsistent with our result in Experiment 3 in which subjects could easily eliminate intermediate movements in the presence of a virtual barrier. While it may be possible to augment mechanistic models with a means to over-rule the generation of intermediate movements where necessary, this raises serious questions about why intermediate movements should ever be permitted. Our alternative interpretation, analogous to previous proposals by Hudson, Landy and Maloney [24], is that intermediate movements reflect a single, deliberate movement plan chosen to maximize performance in the task amid ambiguity about the goal, explaining the presence or absence of intermediate movements without requiring any assumptions about the underlying planning mechanism.

### Limitations of the model

The basic theoretical framework presented here provides a promising unifying framework for describing perception and action. However, the intractability of obtaining the optimal policy for such models is a severe limitation. Recent advances in solution methods for optimal control problems [35] are inapplicable due to the structure of our control problem. Specifically, efficient solution methods require that noise and control act in the same dimensions. The structure of our control problem violates this requirement, since the decision variable has noisy dynamics but is not controllable. The development of efficient numerical methods applicable to the specific class of problems described here would be valuable in generating more precise predictions of the general theory.

Optimal control theory has previously been invoked to account for intermediate movement strategies [36,37]. Such theories suggest intermediate movements as a strategy to exploit execution noise: when multiple, equally valid targets are present, it is better to aim for the middle and let execution noise dictate which target to ultimately hit. Although this theory potentially explains the presence of intermediate movements in the presence of multiple targets, we believe it is insufficient to account for our findings since the amount of execution noise required to predict an intermediate movement given a 90° separation between targets is infeasibly large.

Although our model accurately accounted for the incidence of intermediate movements, one aspect of the data that was not predicted was the fact that the target jump was fully corrected for at about the same delay across all conditions. The model predicts that perfect compensation will be seen earlier under incompatible conditions compared to compatible ones. This discrepancy could potentially be attributable to our model assuming that the pre-jump and post-jump targets should be treated as equivalent, whereas in reality there are important differences between them. The results of Experiment 2 allow us to rule out the possibility that ambiguity about the location of the post-target jump was a major source of asymmetry. It is possible that deteriorating quality of peripheral vision at more eccentric target locations [38] could account for unexpected differences across conditions, although it would be quite a coincidence for this to lead to such close temporal alignment. Additional sources of asymmetry may arise from intrinsic biases in subjects’ decision-making processes; subjects may be inherently biased against changing their minds [34]. Indeed, given that the target jumped in only 30% of trials, it was more likely a priori that the target would remain in its original location.

An alternative explanation for the consistent time at which compensation for the jump became complete is that an underlying mechanistic constraint on movement preparation limited the ability to generate an accurate response to the changed target location. It is unclear how intermediate movements might be reconciled with such a constraint. Nevertheless, our theory provides a rational account of why intermediate movements should ever be allowed to occur, instead of simply always switching abruptly between movement directions.

### Implications for neural representations of movement planning and execution

Our normative model suggests an alternative interpretation of a number of well-established neural correlates of movement planning and preparation. High-level movement goals appear to be represented in dorsal [39] and ventral [40] premotor cortex and also posterior parietal cortex [41]. When multiple potential goals are presented, these goals are represented simultaneously [39,42]. Conventionally, activity associated with a single goal is construed as representing a specific movement plan; simultaneous responses when multiple targets are present is thought to reflect multiple such plans occurring in parallel [23]. Our theory provides an alternative view: that the overall pattern of activity across this population represents a global belief state (a multi-target analog of our binary decision variable *r*_*t*_) over all possible movement goals; details of how to achieve these goals will be determined by a downstream site, possibly primary motor cortex, which is responsible for implementing a single control policy associated with this global belief state.

Computational models have suggested that lateral connectivity within a network representing task goals may provide a mechanism whereby intermediate movements are generated [27,43,44]. Excitatory connections between units tuned to similar movement directions can lead to two peaks of activity becoming merged and thus leading to intermediate movements. These theories therefore explain intermediate movements as a by-product of an underlying planning mechanism. Inhibitory connections between units representing dissimilar movements create winner-take-all dynamics when potential goals are more widely separated. Our results show, however, that intermediate movements are not obligatory; in the presence of a barrier they can be suppressed. This absence of intermediate movements could potentially be explained by inhibition of units representing movement directions that would hit the barrier. However, if it is so easy to eliminate intermediate movements in such a model, it is unclear why they should be permitted in the absence of a barrier.

It is currently unclear exactly how control policies are represented in the brain. However, recent theories have suggested that the state of motor cortex at the time of movement onset is sufficient to encode the full sequence of feedforward motor commands required to execute a movement [45,46]. Typically, neural activity converges onto a movement-specific preparatory state over a period of around 100ms following stimulus presentation [47,48]. It is tempting to interpret this change in neural state as reflecting a form of movement planning. We suggest instead that this observed change in neural state could equally reflect an evolving decision. Indeed, the state of motor areas appears to continuously track belief state during decision-making tasks [49,50]. Although these results are often interpreted as reflecting partially formed or blended motor plans, our theory suggests instead that intermediate states might reflect a single control policy that is optimal given a partially formed belief about movement goals—effectively hedging against possible future fluctuations in belief or changes of mind [34] after the movement has begun.

## Methods

### Ethics statement

All procedures were approved by the Johns Hopkins University School of Medicine Institutional Review Board. All subjects provided written informed consent prior to participating.

### Experimental procedures

24 adult (18–40 y/o, 11 female), right-handed, neurologically healthy subjects were recruited for this study. Subjects were seated at a glass-surfaced table. Their right forearm was supported by a plastic cradle equipped with pressurized air vents to allow frictionless planar arm movements. Subjects' arms were obstructed from view by a mirror positioned above the table surface, through which an LCD monitor (60Hz) displayed movement targets and the position of the index finger in a veridical horizontal plane. The index finger was tracked at 130Hz using a Flock-of-Birds magnetic tracker (Ascension Technology, VT, USA).

A total of 10 subjects participated in Experiment 1. On each trial, subjects were required to position the cursor inside a start circle (10mm diameter). After 300ms, a sequence of four tones spaced 500ms apart was initiated (Fig. 1A). Synchronous with the first tone, a single target (25mm diameter) appeared at one of eight possible target locations, positioned uniformly on a circle of radius 0.08m (Fig. 1B). Subjects were required to initiate a ‘shooting’ movement through the target, synchronous with the onset of the fourth tone. Movement onset was detected based on the first time that the tangential velocity of the cursor exceeded 0.02m/s. In order to be successful, subjects were required to initiate movement within ±100ms of the onset of the fourth tone and move the center of the cursor through some part of the target region. An on-screen graphic displayed peak velocity after each trial and subjects were asked to keep this above a shown threshold that corresponded to 0.9ms^-1^. On successful trials, subjects were rewarded with a “success” tone and points towards a cumulative score. On-screen text following each trial indicated whether subjects had initiated their movement too early or too late. 1s after movement onset, subjects were able to begin the next trial by returning to the start circle.

In an initial familiarization session prior to the main experiment, all subjects received extensive training (>500 trials) at timing their movement initiation accurately. During the main experiment, the target was jumped to a different location in 30% of trials, at a random time (between 150ms and 550ms) prior to the fourth tone. The direction of the new target location differed by either ±45°, ±90° or ±135° from the original (Fig. 1B), and this difference was randomly selected on each jump trial. Subjects performed approximately 2000 trials total, divided into blocks of 100 trials. The full experimental session, including occasional breaks, lasted approximately 3 hours. Some subjects performed the main experiment across two separate sessions on different days.

Experiment 2 followed the same pattern as Experiment 1, except that there were only 2 potential target locations. Six new subjects (4 Female) performed 500 trials (across 5 consecutive blocks) with a 45° separation between potential target locations (±22.5° relative to straight-ahead), and 500 trials with a 135° separation (±67.5° relative to straight-ahead). The order of target configurations (45° separation first or 135° separation first) was counterbalanced across subjects.

The same basic setup was used in Experiment 3. Eight subjects participated in this experiment (3 Female), two of whom had also participated in Experiment 1. Subjects performed two main sessions, each consisting of 8 blocks of 100 trials. The *No Barrier* session was similar to Experiment 1, with 8 potential targets and the target jumping on 30% of trials, except that all jumps were ±45° in magnitude. The *Barrier* session was identical to the *No Barrier* session, except that a series of virtual barriers was introduced in the workspace (Fig. 5A). These barriers encouraged subjects to move in a straight line towards each target and prohibited intermediate movements. The barrier configuration effectively created a 10mm wide channel within which subjects could move freely. On trials in which subjects entered the barrier region, the barrier turned red, an unpleasant tone was played, and the subject received no score on that trial. Subjects were also verbally encouraged to avoid contacting the barrier. All subjects performed one session with barriers present and one session without and the order of sessions was counterbalanced across subjects. Barriers were also present during the second half of the initial familiarization session in which there were no target jumps.

### Data analysis

Position and velocity data were smoothed using a 2^nd^-order Savitzky-Golay filter with half width 54ms. We computed the time of movement onset based on the latest time that the smoothed tangential velocity was less than 0.02m/s prior to the peak tangential velocity (note that this differed slightly from the onset time calculated online that determined the success or failure feedback given to subjects about the timing of their movements during the experiment). We expected that the initial direction of movement would depend on the amount of time available to revise the movement plan prior to movement initiation. We therefore computed, for each trial, the re-preparation time (rPT)—the duration between the time of the target jump and the time of movement onset. We computed the initial reach direction based on the direction of the tangential velocity 100ms after movement onset. All jump trials were transformed into a common reference frame such that the initial target was located at 0°, and the target jumped in a positive direction. Trials in which the hand failed to move further than 5cm from the start location were excluded from the analysis.

In a small number of trials, movements were excessively curved in a way that did not permit a well-defined estimate of the reach direction 100ms after movement onset. This was often associated with failure to keep the hand stationary prior to movement initiation. We identified and eliminated excessively curved movements as follows: we computed the rate of change of estimated movement direction with respect to measurement time,

$$\frac{d\hat{\theta }}{dt}=\frac{\hat{\theta }(100+\Delta )-\hat{\theta }(100-\Delta )}{2\Delta },$$ (1)

where $\hat{\theta }(t)$ is the estimated reach direction at time *t* and Δ=1130 ms. Based on behavior in non-jump trials, we set a threshold on the absolute value of this rate of 1.3°/ms. Of all non-jump trials, 99% fell within this range. As a result of this exclusion procedure, an average of 4.5±3.1 trials per subject were excluded in Experiment 1. These excluded trials were distributed similarly across each of the possible jump types (F(2,18) = 3.15; *p* = 0.07). An average of 4.7±3.3 trials per subject were excluded in Experiment 2, and an average of 2.75±3.0 trials per subject were excluded in Experiment 3, also not depending on the condition (Experiment 2, F(1,5) = .03; p = 0.8; Experiment 3, F(1,7) = 0.02; *p* = .88). In total, we excluded less than 3% of all trials on the basis of excessive curvature.

In order to quantify the timecourse of the change in initial reach direction for comparison across conditions and across subjects, we assumed that the initial reach direction followed a sigmoidal relationship with available re-preparation time:

$$\theta =S(rPT)=\frac{A}{1+e^{-\frac{\left(rPT-t_{50}\right)}{\tau }}}.$$ (2)

We assumed that *A* was equal to the actual jump amplitude. This function therefore contained two free parameters: a slope parameter *τ* that characterized the timescale over which gradual changes in reach direction occurred, and a latency parameter *t*_50_ which acted to shift the sigmoid along the time axis. An important feature of the data is the presence of uncertainty not just in the estimated reach direction, but also in the estimated rPT. In the presence of such uncertainty, an ordinary least squares fitting approach significantly overestimated *τ*. We therefore adopted a maximum likelihood approach that specifically accounted for the uncertainty in the rPT. Specifically, we assumed Gaussian noise both in the reach direction, due to either execution variability or measurement noise (s.d. *σ*_*θ*_), and in the estimated re-preparation time, due to either variability on the part of the subject, uncertainty in our estimate of the movement onset time, or experimental error in controlling the precise target presentation time (s.d. *σ*_*t*_). The likelihood for each observation was consequently given by

$$L_{i}\propto \int \exp\left[-\frac{{\left(e-t\right)}^{2}}{2\sigma_{t}^{2}}-\frac{{\left(\theta_{i}-S(e;t_{50},\tau )\right)}^{2}}{2\sigma_{\theta }^{2}}\right]de,$$ (3)

where *e* reflects possible values for the noise in the measured value of the rPT. We set *σ*_*θ*_ equal to the mean standard deviation of initial movement directions on non-jump trials across all subjects (*σ*_*θ*_ = 10.7). We set *σ*_*t*_ = 10 ms since this value was found to lead to robust performance on pilot data. This likelihood was evaluated by trapezoidal integration over *e*.

Not all subjects consistently generated data with rPTs in the critical slope region of the sigmoid. Behavior for which this data was unavailable was thus equally consistent with a broad range of sigmoid parameters. We resolved this ambiguity by biasing the sigmoidal fit towards more shallow slopes through an additional term added to the log-likelihood:

$$\Lambda =\sum_{i}L_{i}+\alpha \tau .$$ (4)

Thus the estimated slope was the shallowest (longest duration) slope that was consistent with the data. Importantly, this approach was conservative since this ambiguity tended to occur during larger amplitude target jumps where behavior was expected to be more abrupt. We set *α* = 0.02, based on fits to synthetic data.

Finally, maximizing this likelihood yielded accurate parameter estimates on synthetic datasets, but was quite sensitive to outlying data points in real data. We therefore extended our parameter estimation procedure to make it more robust to outlying data points by supposing that each data point could have been generated by an alternative, uniform distribution, with a fixed likelihood *L*_0_.

We identified the sigmoid parameters that maximized the likelihood of this mixture model using an expectation maximization algorithm [51,52]. Following this procedure, we rejected an average of 3.9%±2.3% of data points per subject from Experiment 1 as outliers and 1.7±1.5% of data points in Experiment 2, in neither case biased towards any particular condition (p>0.05). In Experiment 3, the average outlier rejection rate across subjects was 3.2%±1.9% and was marginally but consistently greater in the barrier condition (2.5%±1.8% No Barrier, 3.9%±1.8% Barrier; F(1,7) = 5.61; p<0.05).

### Optimal action selection amid evolving uncertainty about task goals

Here, we consider the problem of selecting optimal actions in order to achieve a goal in the presence of uncertainty. We model the arm through a linear dynamical system in discrete time with state **x**_*t*_, and subject to time-varying controls **u**_*t*_:

$$x_{t+1}=Ax_{t}+Bu_{t}.$$ (5)

We characterize the goal of the task through an accuracy cost *J*_**x**_ that penalizes deviations from some goal state **g** at the end of the movement (time *t* = *T*). In addition to this accuracy cost, we assume an effort cost *J*_**u**_ that penalizes large motor commands. It is difficult to say *a priori* exactly what the form of this cost should be [53]. Following standard approaches [20], however, we assume that this effort cost is a quadratic function of the overall sequence of motor commands:

$$J_{u}=\sum_{t}w_{t}u_{t}^{2}.$$ (6)

In this equation, *w*_*t*_ is a potentially time-varying weight. According to the optimal feedback control hypothesis [20], the motor system selects motor commands **u**_*t*_ that minimize the sum of accuracy and effort costs:

$$J=J_{x}(x_{T}-g)+J_{u}.$$ (7)

The key novelty of our model is that the location of the goal state **g** is not precisely known. Specifically, we assume that the true goal is at one of two possible locations, **g**_1_ and **g**_2_. Supposing that *p* represents the belief that **g**_1_ is the true goal location, and (1−*p*) the corresponding belief for **g**_2_, we introduce an evidence variable *r* which reflects the perceived log-odds ratio between the two targets:

$$r=\log\left(\frac{p}{1-p}\right).$$ (8)

Furthermore, we assume that the belief about the state of the target can vary over time. As is commonly assumed in decision-making models [3,26], we model *r*_*t*_ as following a Gaussian random walk: $r_{t+1}∼N(r_{t},\sigma_{r}^{2})$. In models of decision-making, the stochastic nature of *r*_*t*_ reflects a distribution over stimuli that the subject may have perceived in a given trial. In this case, however, the dynamics of *r*_*t*_ reflect the subjects’ subjective prior expectations about how their belief might change in the future, after movement onset. We set *r*_*t*_ to follow a random walk with zero drift, reflecting the fact that subjects should expect their beliefs to change, but are not biased to expect that they will change in any particular direction. Note that we do not attempt to explicitly model the actual evolution of *r*_*t*_ through the movement in response to presented stimuli. Instead, we focus on the implications that the possibility of future evidence being accumulated during the movement will have for the choice of action at the start of the movement.

The overall expected cost depends upon the ultimate belief about the target location, *r*_*t*_, at the end of the movement (time *T*), i.e. *r*_*T*_:

$$\begin{array}{c} E[J]=p_{T}J_{x}(x_{T}-g_{1})+\left(1-p_{T}\right)J_{x}(x_{T}-g_{2})+J_{u}\\ \;\;\;\;\;=\frac{1}{1+e^{-r_{T}}}J_{x}(x_{T}-g_{1})+\frac{1}{1+e^{r_{T}}}J_{x}(x_{T}-g_{2})+J_{u}. \end{array}$$ (9)

Our hypothesis is that subjects act to minimize the expected value of this cost. Solving this optimal control problem is not straightforward. Although the overall state of the system (combining the limb state **x**_*t*_ and belief state *r*_*t*_ into a single vector) has linear dynamics and Gaussian noise, the endpoint cost is a non-linear function of that state (Equation 9). This precludes usual solution methods for optimal control problems which require an endpoint cost that is quadratic in the state. We are therefore forced to rely on a dynamic programming approach [54] which severely limits the dimensionality of problems for which we can obtain a solution.

We implemented a simplified model to demonstrate the key features of behavior predicted by this framework. We modeled the center-out reaching task with a single spatial dimension *x*_*t*_ representing the angular position of the hand. We assumed that the motor command *u*_*t*_ specified the instantaneous angular velocity of the hand, i.e. ${\dot{x}}_{t}=u_{t}$. We assumed a fixed time horizon of 200ms. We set *w*_*t*_ in Equation 9,8 to increase linearly from 0 at *t* = 0 to *w*_*MAX*_ at *t* = *T*, to reflect the fact that achieving a given angular velocity requires a higher Cartesian velocity when the hand is further away from the start position and should therefore be more costly. The endpoint cost for each target was given by a step function with width *a* around the goal region.

Jx(x)={0−a2<x<+a21otherwise. (10)

In order to determine the control policy that minimized the total expected cost (Equation 9) we discretized the state space (angular position discretization of 0.1°, belief discretization of 0.5, and time discretization of 10ms) and used dynamic programming [54] to find the optimal expected cost-to-go *V*(*x*,*r*,*t*) at each state and time. We used the value function at *t* = 0 to determine the optimal initial reach angle *x*_0_* for each possible initial belief *r*_0_, i.e.

$$x^{*}\left(r_{0}\right)=\arg\underset{x}{\min}\left\{V\left(x,r_{0},0\right)\right\}.$$ (11)

We manually selected model parameters (*w*_*MAX*_ = .001, *a* = 1, *σ*_*r*_ = 1) that yielded qualitatively similar predictions to actual subject behavior. Note that our aim here was not to provide a quantitative fit to the data but to demonstrate the feasibility of our theory to account for our observations—principally the interaction between target separation and the time course of intermediate movements.

Finally, based on observed discrepancies between the data and the model (see Results and Discussion), we considered the possibility that the nature of the task may have created an asymmetry between the initial goal and the post-jump goal that is not captured in the basic form of the model. We accommodated some asymmetry within the model through an asymmetric cost function in which the cost function *J*_**x**_ for the initial target location was scaled relative to the post-jump target location:

$$J=\alpha_{1}p_{t}J_{x}^{1}(x-g_{2})+\left(1-p_{t}\right)J_{x}^{2}(x-g_{2})+J_{u}.$$ (12)

We set *α*_1_ = 10 in order to yield behavior that qualitatively matched observed behavior.
